# Forward effects from action observation: the role of attentional focus

**DOI:** 10.1007/s00426-023-01888-8

**Published:** 2023-10-26

**Authors:** Francesco Ianì, Teresa Limata, Ivan Nabil Ras, Monica Bucciarelli

**Affiliations:** 1 Dipartimento di Psicologia, Università di Torino, Via Verdi, 10, 10124 Turin, Italy; 2 Centro di Logica, Linguaggio, e Cognizione, Università di Torino, Turin, Italy

## Abstract

After viewing an image representing an action on an object, we recognize the forward states of the seen action faster than the backward states. The present study exploits a variant of a new experimental paradigm to investigate cognitive mechanisms underlying this effect. Participants viewed a series of still photos of unfolding actions on objects, each followed by a photo depicting either one of three (instead of two of the original paradigm) different and temporally distant moments after the image or one moment before the image, along with photos of different actions. Experiment 1 revealed the classical forward effects in this new context: when the task was to judge whether the action in the second photo was the same as in the first photo, evaluations were faster for all forward photos than for backward photos. In Experiment 2, we examined the role of participants’ attention to the object alongside the role of attention to action kinematics in triggering these “forward effects” by manipulating participants’ attentional focus. As the results showed, evaluations were faster for all forward photos when the focus was on the action kinematics, but when the focus was on the object, evaluations were faster only for the last forward photo showing the final action state. These results seem to suggest that focusing on the object triggers a representation of the action goal and thus modulates the mental simulation underlying action anticipation.

## Introduction

Both the observation of an action and the observation of a still image of an action trigger a mental representation of the action forward in time (e.g., Chen et al., 2021; Wilson & Knoblich, 2005). This cognitive process is fundamental to effective interaction with the environment, but it can also lead to perceptual errors, i.e., distorted representations of a moving target that is shifted in the direction of implied motion. This potentially negative effect was first demonstrated in studies of the so-called representational momentum, although this literature did not focus exclusively on human action (e.g., Freyd & Finke, 1984). In representational momentum paradigms, participants typically first look at a photograph showing an actor or object in motion. Then, the photo disappears and a new photo appears on the screen and their task is to judge whether it is exactly “the same” or “different from” the first one. As the results of a study revealed, they found it harder to reject distractors when they were photos of the same scene shot later in time than when they were photos shot earlier in time (e.g., Freyd, 1983). It has been argued that this “forward effect” is due to the mental representation of the action being shifted along the pattern of motion implied in the photo (for a review, see Hubbard, 1995, 2005). Consistent with this claim, eye movement studies have shown that participants tend to make anticipatory saccades toward the target positions of the observed movements when watching action videos (Flanagan & Johansson, 2003).

Recently, Ianì (2021) developed a new paradigm to investigate forward effects in action observation. In one such study, participants watched a series of videos, each showing the central part of an action performed by an actress on an object, such as the actress eating a hamburger (Ianì et al., 2021). Shortly after each video, they were shown a photograph depicting either the beginning or the end of the entire action, i.e., states of the action that were not seen in the video. Their task was to judge whether the photo showed something that happened before (backward photo) or after (forward photo) the action shown in the video. The results revealed that forward photos were assessed faster than backward photos. This suggests that people represent the observed action unfolding in time and that this representation speeds up the recognition of the next action states compared to the backward ones (Ianì et al., 2021). A series of studies detected this forward effect with three different versions of the task, in which participants observed photos rather than videos during encoding. In two versions of the task (Experiments 1 and 2 in Ianì et al., 2023), participants viewed pairs of photos representing the first and last stills of the central part of an action video; they then viewed a second photo showing the same action, but either forward or backward in time. Participants were asked to judge whether the second photo showed an action phase that occurred before or after the action phase observed in the first pairs of photos (explicit task in Experiment 1): forward photos were assessed faster than backward photos. The same effect was found with a “picture–picture verification task” similar to the so-called “sentence–picture verification task” (e.g., Zwaan et al., 2002; Borghi et al., 2004). When the participants were asked to judge whether the second photo depicted the same action seen in the first pair of photos without being explicitly asked to consider the direction of the action (implicit task in Experiment 2), they were faster when the second photo was a forward photo compared to when it was a backward photo. In another version of the task (Experiment 3 in Ianì et al., 2023), at encoding, participants observed just one still frame extracted from the middle of action videos and then, at test, they were invited to decide whether the photo presented depicted a scene congruent or not with the action seen in the photo at encoding (implicit task from single still frame). At test the participants encountered two types of backward photos, depicting the action at two different temporal distances before the central part of the action, and two different types of forward photos, depicting the action at two different temporal distances after the central part of the action. As the results showed, the reaction times were shorter for both forward photos, regardless of temporal distance. This suggests that forward effects not only affect the nearest state of the action, but also states of the action to come.

In the present study, we used three different forward states of actions instead of the two used in Ianì et al. (2023) to investigate the role of the participants’ attention to the object involved in the action in modulating the forward effect.

## The role of action and object in anticipation

The cognitive-experimental literature supports the claim that action anticipation may be based on a mechanism of action prediction in which people simulate the next states of the observed action through a mental sensorimotor simulation (a representation of the action unfolding in time) that is likely based on motor activations. Several studies suggest that different intentions lead to different kinematic patterns (e.g., Sartori et al., 2009) and that the kinematic features of a given action allow the observer to predict its end and goal (Castiello, 2005). Consistent with these assumptions, the motor information conveyed in action images or videos is sufficient to identify the intention of the actor in question (see e.g., Manera et al., 2015) or the fate of the action (e.g., whether a basket shot succeeds or fails, Aglioti et al., 2008). In other words, people use subtle motion cues from the observed actor to predict the end of the action (e.g., Stapel et al., 2012). For example, people can predict whether a movement for reaching for a bottle will be completed to pour out the water or to drink directly from the bottle, or whether a particular movement will be performed with cooperative or competitive intentions (e.g., Manera et al., 2015). Observers use a subset of discriminative kinematic features across the entire kinematic pattern to identify intention from the observation of simple motor actions. These kinematic variables are physical features of the movements (in a reaching movement, e.g., speed, grip aperture, the eyes’ fixation, etc.). Some of these kinematic cues are also reflected in photos of actions, and thus can support the recognition of the temporal “direction” of an action based on a single screenshot (see e.g., Urgesi et al., 2006; Kislinger, 2021). This literature suggests that participants simulate the observed action step by step through a process of “soft motor resonance” to identify an intention from a motor information (Ianì, 2021).

At the neurocognitive level, a meta-analysis of 104 neuroimaging studies on action observation has shown the activation of the so-called action observation network (hereafter AON network) during the observation of both object-related and non-object-related actions (Caspers et al., 2010). Specifically, in studies in which participants observed object-related and non-object-related actions with different effectors, namely mouth, hand, and foot (Buccino et al., 2001) or only non-object-related actions (Urgesi et al., 2006), activation of mirror neurons in premotor areas was found. Further, Urgesi et al. (2010) found that motor activation is greater when participants observe ongoing and incomplete actions than when they observe completed movements. Thus, these studies suggest the importance of the AON network in the action prediction mechanism and support the existence of an “embodied” mechanism underlying action anticipation.

However, one should not interpret this mechanism as the only one at stake in action prediction. There are several studies suggesting that multiple dimensions (e.g., the environment constraint as well as the social and emotional context, e.g., Edwards et al., 2022; McDonough & Bach, 2023) may influence cognitive mechanisms triggered by action observation. Specifically, the characteristics of the object involved in the observed action play a special role (see, e.g., Azaad & Laham, 2019). Indeed, a meta-analysis of neuroimaging studies suggests that activation of the AON network varies depending on whether participants observed non-object-related actions or object-related actions (Reynaud et al., 2019). Observing actions with objects recruits non-motor neural resources, specifically area PF in the left inferior parietal lobe (IPL), to identify appropriate mechanical actions through technical reasoning (Osiurak et al., 2020). The IPL appears to support the cognitive ability to reason about the physical properties of objects, so that humans can, for example, choose among several objects the one that is best suited for executing a particular goal (see, e.g., Goldenberg & Spatt, 2009). Also experiments using the so-called “violation paradigm”, in which an object is used with the wrong kinematics, seem to indicate how the mere object’s features can help observers make predictions about possible action goals (Decroix & Kalenine, 2018). They also show early and initial activation of goal representations linked with the object involved in the action (Van Elk et al., 2008).

Knowledge about the functions associated with objects is a kind of top-down knowledge that can influence action prediction by modulating mental simulation. Studies have already shown that top-down knowledge, such as about an actor’s intention, can modulate action anticipation. For example, in one study, participants heard an actor declare an intention to either take or leave an object and then saw the actor either reach for the object or withdraw from it, such that action and intention were either congruent or incongruent (Hudson et al., 2016). Consistent with the results of studies conducted under the representational momentum paradigm, observers generally perceived the point of hand disappearance to be farther along the trajectory than it actually was, and this effect was larger for actions that were congruent with the actor’s stated intention. These results suggest that action prediction integrates both current motion and top-down knowledge of the actor’s intention. We hypothesize that top-down knowledge about the object feature can also modulate action anticipation when the observer focuses on the object of an action.

As noted above, over the past decade, studies of action observation often concerned object-related actions (e.g., eating a hamburger). However, few studies have attempted to detect a possible modulating role of the attentional focus in action anticipation. Among them, studies have shown that attention can be focused on different aspects of the actions of others, such as the hands of the doer or the object involved in the action. Flanagan and Johansson (2003) showed that the presence of an object involved in another action is a determining factor in whether observers’ eye movements are predictive or reactive. When observers saw an action on an object with a specific target, their eye movements were more predictive, anticipating the subsequent phases of the action. This suggests that observers activate motor representations similar to those used in the execution of the action itself. In contrast, the eye movements of observers who saw an action that did not have a specific purpose or did not involve object manipulation tended to be reactive, that is, they simply followed the motion of the object or events in the environment without actively anticipating the subsequent steps. The results of a study by Hamilton and Grafton (2006) support the idea that individuals who observe others performing actions focus more on the kinematic features of the actions, such as how the actions are performed, rather than fixating exclusively on the objects involved. The researchers found increased activation in brain regions associated with motion perception and action-related information, suggesting that participants’ attention was predominantly focused on the kinematic features of the observed actions rather than exclusively on the objects involved.

## The present study

**Fig. 1:**
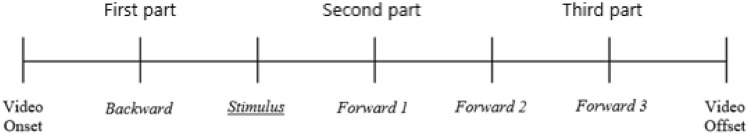
Method for extracting photos from each action video

The present study aims at a deeper understanding of the role of the attentional focus on action and on object in action anticipation. To this end, participants in the present study viewed still images of actions extracted from the same videos as in Ianì et al. (2021). Each original video showed a single action performed by an actress using her upper limbs, either one or two arms (see the full set in the online Supplementary Material). For the purposes of the present investigation, each video was cut into three parts of equal length (three video parts). The first frame of the second part was presented to participants at test (stimulus photo). For each action video, four additional photos were extracted: the central image of the first part (backward photo), the central image of the second part (Forward 1 photo), the last image of the second part (Forward 2 photo), and the central image of the third part (Forward 3 photo) (see Fig. 1).

After viewing each stimulus, participants were presented with test stimuli representing a moment after (one of the three forward photos) or before (the backward photo) the stimulus, as well as photos representing different actions. Thus, unlike in the studies of Ianì and colleagues (2021; 2023), participants in the present study saw three different forward photos, a difference that allowed us to test the prediction that focusing on the object triggers a representation of the action goal and thus modulates the representation of the action unfolding in time, i.e., the kinematic mental simulation.

In Experiment 1, participants had to judge whether the second photo depicted a scene congruent with the action shown in the first photo. We clarified to participants that “congruent” referred to whether the test photo represented the same action or a different action than the one in the stimulus photo, and participants were given examples. For example, they were told that if they see an actress taking a violin from her box in the first picture, and the same actress playing a violin in the second example, the answer is “yes”. On the other hand, if the actress in the second photo picks up or plays a guitar, the answer is no. If the forward effects found in the previous studies are genuine, participants should respond faster to all forward photos than to the backward photo. In Experiment 2, participants’ task was the same, but we also manipulated participants’ attentional focus. One-half of the participants were told that in judging whether the second photo depicted a scene congruent with the action shown in the first photo, they had to consider whether the second photo showed the same action as the first photo (action-focused group), whereas the other half were told that they had to consider whether the second photo showed the same object as the first photo (object-focused group). If focusing on the objects modulates the representation of the action unfolding in time, then we should expect a significant interaction, i.e., the forward effect should operate differently in the two groups. While participants in the action-focused group should show an advantage (in terms of RT) for all forward photos, participants in the object-focused group should show an advantage only for the last forward photo, representing the final action state (the action goal).

## Experiment 1

Participants in the experiment saw a series of stimulus photos and, shortly after each photo, a second photo, either one of the three forward photos or the backward photo. Their task was to judge whether the second photo depicted a scene that was congruent with the action shown in the first photo, i.e., whether the action was the same or not. The prediction was that participants should respond faster to all forward photos than to the backward photo.

## Method

### Participants

A power analysis revealed that at least 14 participants were required to obtain a suitable statistical power level of 0.95 to detect a significant effect and assuming a medium effect size (*f* = 0.25; like those obtained in Ianì et al., 2023), a high mean correlation among repeated measures of 0.83 (as obtained in Ianì et al., 2023) and with *α* = 0.05. Participants were 27 students from the University of Torino (6 males and 21 females, mean age = 27.37; SD = 7.91). They voluntarily took part in the experiment after signing an informed consent form.

### Materials

**Fig. 2:**
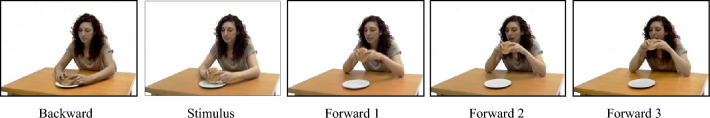
The images extracted from the video “Eat a hamburger” and presented to participants at encoding (stimulus) and at test (Backward, Forward 1, Forward 2, Forward 3)

The materials consisted of 14 videos from a previous study of Ianì et al. (2023). Each video lasted about 3 s and showed a single action performed by an actress using her upper limbs, either one or two arms (e.g., answering the phone; see the description of the stimuli in Appendix A, Table 4). We chose these actions because they are common in everyday contexts and can be performed while sitting and interacting with an object. Each video was cut into three parts of equal length, from which photos were extracted, as described in “The present study”. Figure 2 shows examples of the stimuli obtained for the video “Eat a hamburger”. We used the photos of seven videos as targets, whereas the photos of the other seven videos were used as filler stimuli at test.

Some actions we have used as experimental material, such as those we often observe in the natural environment, involve not only the acquisition of an object for use, but also an accompanying reverse action in which the object is returned (“return” actions). For instance, this is true for the action “Drink a glass of water” in which the person usually puts the glass back on the table after drinking it. Other actions instead do not involve a return phase with the same object. For example, the scenarios “Peel a banana” or “Tear a sheet of paper” do not involve a return phase (“no-return” actions). In the case of return actions, what we call a backward action could in principle be the moment furthest forward in time (in the hamburger example, the hamburger on the plate is now considered backward, but could be the fourth forward, as an anonymous reviewer argued). In a preliminary experiment of our previous studies (Ianì et al., 2023), which included the same material as the present study, we ensured that the direction of the action was detectable from the action photos. For each series of five photos of a single action, 12 participants viewed the third, central photo and were asked to rate each photo as forward or backward. Results revealed that they were almost always accurate in recognizing both the forward photos as forward actions (97%) and the backward photos as backward actions (90%), with all participants performing better than chance (binomial test, *p* always < 0.007). This seems to suggest how, by just observing a single picture, participants were able to interpret the correct direction/goal of the action. In other words, the kinematic features of the photo showing a hamburger held in the hands near a plate differ whether it is the beginning of the action of picking up the hamburger or the action to put the hamburger on the plate. In addition, we also investigated possible differences in accuracy and reaction times between return and no-return scenarios among participants in our first experiment.

### Design, task and procedure

We used a within subjects design with Types of photo (Backward, Forward 1, Forward 2, Forward 3) and Type of action (Return, No-return) as within-subjects factors. At test, each stimulus was combined with the four test photos depicting the action forward or backward in time and with four filler photos depicting actions other than those shown in the photo at encoding. The test stimuli were 50% photos representing a congruent scenario (i.e., requiring “Yes” responses) and the other 50% photos representing a different scenario (filler photos requiring “No” responses). This was done to achieve a balance of yes and no trials and to keep participants’ attention focused on the task throughout the section. For each participant, the stimuli were presented in a random order, with the only constraint that the same scenario was not presented in two consecutive trials (e.g., the Backward photo and the Forward 2 photo of the hamburger scenario). In total, each participant performed 56 trials. The experiment took place online. Participants received an email with the instructions to download E-prime Go (Psychology Software Tools, 2020), an extension of the software used to conduct online experiments. Participants received the following instructions through a computer screen:

Thank you for participating in this experiment on how we understand the unfolding of an action over time. You’ll see a photo that represents an action in progress. Soon after each photo a central fixation cross will appear, followed by another photo. Your task is to decide whether the photo depicts a scene congruent or not with the action seen in the initial photo. Answer out loud by saying YES if you think it is congruent, by saying NO if you think it is not.

It was pointed out that the fixation cross was very fast and it was necessary to pay attention to it because a photo would appear right after it. It was also specified that the term “congruence” referred to whether the action in the two photos was the same or different (i.e., there was no reference to the direction of the action). When participants were ready to begin, they pressed the space bar and the first stimulus photo appeared on the screen for 3 s, followed by a fixation cross of 250 ms duration. Then, the test photo appeared on the screen for 7 s. During this time, participants were asked to respond verbally. This interstimulus interval (ISI) granted that participants had to rely on simulation mechanisms; indeed, ISIs shorter than 150 ms generate apparent visual motion that relies on perceptual mechanisms (Verfaille & Daems, 2002). The test photo was followed by a white screen with the text “Next photo” lasting 3 s. Participants were asked to give vocal responses to avoid classic manual responses (e.g., key presses). Asking participants to respond manually implies the use of the motor resources of the hands/arms that may be at play in processing the observed action (e.g., Ianì, 2021).

### Data recording

We coded as accurate YES responses to photos (both forward and backward) showing the same action as in the initial photo, and NO responses to photos not showing the same action as in the initial photo. Responses to filler trials were not considered in the analyses. We measured participants’ response times with MatLab 2020 software (The MathWorks Inc., 2020), from photo presentation to participants’ oral answer. Specifically, RTs were measured by calculating milliseconds from photo presentation onset to oral answer onset (the Matlab script used to calculate RTs is available online in the Supplementary Materials). We chose this solution, compared to measuring milliseconds from photo presentation to oral answer ending, to avoid potential differences in the lexical processing required to say “YES”/“NO” (“sì”/“no” in Italian). The Matlab script calculates the medium noise of the entire 7 s given to participants to respond and sets a threshold for each trial. If the noise exceeded the threshold, RT was calculated.

## Results

The results of 2 of the 27 participants were dropped from the analysis because their accuracy rate was 2.5 standard deviations below the mean. We report the statistical analyses for the remaining 25 participants. Table 1 shows the mean response times and mean accuracy for correct evaluations for each type of photo.

**Table 1:** Mean RTs and mean accuracy (and standard deviations) for correct evaluations of each type of photo in Experiment 1

	Types of photo			
	Backward	Forward 1	Forward 2	Forward 3
RTs	1326.55 (326.71)	1159.14 (356.48)	1150.25 (325.64)	1108.099 (311.19)
Accuracy	0.91 (0.14)	0.96 (0.07)	0.99 (0.04)	0.94 (0.11)

We conducted an analysis on log-transformed reaction times (hereafter, RT) for correct responses. Since the log-transformed RT did not violate the normality assumption, we performed a 4 × 2 repeated measures ANOVA with Types of photo (backward, Forward 1, Forward 2, Forward 3) and Type of action (return vs. no return) as within-subject factors on reaction times. We did find a main effect of the Types of photo (*F*(3,72) = 8.27, *p* < 0.001, *η*_p_^2^ = 0.25), whereas both the main effect of Type of action (*F*(3,72) = 2.67, *p* = 0.12, *η*_p_^2^ = 0.1) and their interaction (*F*(3,72) = 0.37, *p* = 0.77, *η*_p_^2^ = 0.02) were not significant. Post hoc comparisons (*p* values Bonferroni adjusted) revealed significant differences between the evaluation of backward photos and all types of forward photos. Specifically, reaction times of backward photos were slower compared to Forward 1 photo (*p* < 0.05, CI [11.8, 314.2], Cohen’s *d* = 0.62), Forward 2 photo (*p* < 0.05, CI [23.0, 313.0], Cohen’s *d* = 0.67) and Forward 3 photo (*p* < 0.01, CI [52.5, 347.4], Cohen’s *d* = 0.78). The comparisons between the forward photos were not significant (*p* always = 1, BF_01_ varied from 2.33 to 4.72^1^). Thus, the advantage in the evaluation of forward photos occurs for every type of forward photos, regardless of the distances between those forward photos and the photo presented at encoding.

The analyses on accuracy rates did not reveal significant effects. Because the normality assumption was violated, using the RM function in the MANOVA.RM package (Friedrich et al., 2018) in the R statistical programming environment (version 4.1.0; R Development Core Team, 2021), we extracted the ANOVA-type statistic (ATS) with the corresponding *p *values, as this test is expected to perform well even if the normality assumption is violated (Friedrich et al., 2018). We found no effect of Types of photo (ATS = 2.98, *p* = 0.06), no effect of Type of action (ATS = 1.03, *p* = 0.31), and no significant interaction (ATS = 0.92, *p* = 0.41). Thus, participants were not more accurate in any of the experimental conditions.

To rule out the possibility that these results could be explained by perceptual similarities between the photos, we computed an objective index of perceptual similarity between test photos and stimuli photos using the FSIM algorithm (Zhang et al., 2011). The closer the FSIM index is to 1, the more similar the images are. FSIM indexes did not vary across photos (see Table 2). We found no significant effect of the Types of photo (Greenhouse–Geisser correction, *F*(1.519, 9.115) = 2.93, *p* = 0.11, *η*_p_^2^ = 0.33), ruling out a possible role of perceptual similarity.

**Table 2:** FSIM perceptual indexes for all test photos as a function of the scenario

Scenario	Backward stimulus	Forward 1 stimulus	Forward 2 stimulus	Forward 3 stimulus
Banana	0.9217	0.8994	0.9006	0.8986
Glass	0.9382	0.9419	0.9553	0.9507
Hat	0.9093	0.8742	0.8891	0.8836
Nose	0.8960	0.9072	0.8993	0.8943
Flower	0.9400	0.9286	0.9348	0.9299
Sheet	0.9395	0.9286	0.9343	0.9282
Hamburger	0.9224	0.9147	0.9180	0.9098
**Means**	**0.9239**	**0.9135**	**0.9188**	**0.9136**

## Experiment 2

The experimental task was the same as in Experiment 1, except that we manipulated participants’ attention with further instructions. In judging whether the second photo depicted a scene congruent with the action shown in the first photo, participants in the action-focused group were instructed to pay attention to whether or not the action in the second photo showed the same action as the first photo, whereas participants in the object-focused group were instructed to pay attention to whether or not the second photo showed the same object as the first photo. The prediction was that participants in the action-focused group should be faster in judging all forward photos than in judging the backward photo, and participants in the object-focused group should be faster only on the last forward photo.

## Method

### Participants

The participants were 100 students of the University of Torino (48 males and 52 females, mean age = 27.52, SD = 8.53). A power analysis revealed that at least 38 participants for each group were required to obtain a suitable statistical power level of 0.95 to detect a significant effect and assuming a small effect size (*f* = 0.1), a high mean correlation among repeated measure of 0.83 (as obtained in Ianì et al., 2023) and with *α* = 0.05. The participants, who voluntarily took part in the experiment, signed the informed consent before the experiment.

### Material, design, task and procedure

The material was the same as that in Experiment 1. We used a mixed design with Attentional focus as between-subjects factor (action focused, object focused) and Types of photo as within-subject factor (backward, Forward 1, Forward 2, Forward 3). At test, each stimulus was combined with the four test photos depicting the action backward or forward in time and with 4 filler photos depicting different actions from those portrayed in the photo seen at encoding. For each participant, stimuli were presented in a random order, with the only constraint that the same scenario was not presented in two consecutive trials. Overall, each participant performed 56 trials. The experiment took place online. Half of the participants were assigned to the action-focused group and the other half was assigned to the object-focused group. Participants received the following instructions:

Thank you for participating in this experiment on how we understand the unfolding of an action over time. You’ll see a photo that represents an action in progress. Soon after each photo a central fixation cross will appear, followed by another photo. Your task is to decide whether the photo depicts a scene congruent or not with the action seen in the initial photo.

At this point, instructions differed for the two groups:

By congruent we mean whether the *action performed by the actor* in the two photos is the same or not (Action-focused group).

By congruent we mean whether the *object* in the two photos is the same or not (Object-focused group).

The final part of the instructions was identical for the two groups:

Answer out loud by saying YES if you think it is congruent, by saying NO if you think it is not.

The procedures for presenting the stimuli were the same as in Experiment 1.

### Data recording

The measurements of accuracy and RTs were the same as in Experiment 1.

## Results

**Fig. 3:**
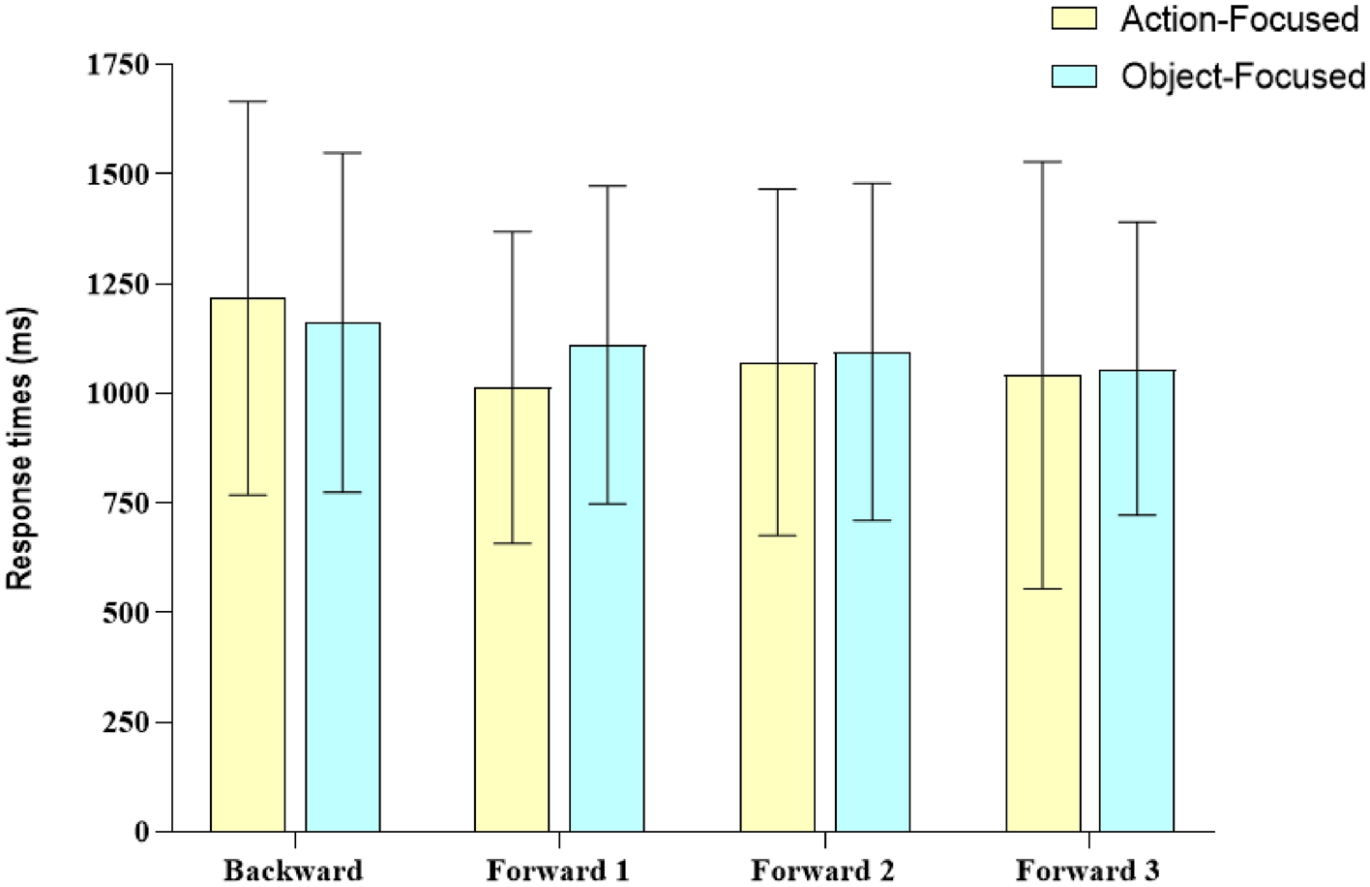
Responses times (in ms) and standard deviations for correct evaluations based on both Attentional focus and types of photo factors in Experiment 2

Data from eight participants were excluded from the analysis because their accuracy rate was 2.5 standard deviations below the means (the data of 2 outliers in the object-focused group and the data of 6 outliers in the action-focused group). Thus, we analyzed the data of the remaining 92 participants. Figure 3 shows the mean RTs for correct evaluations based on both Attentional focus and Types of photo factors. We conducted a mixed repeated measures ANOVA with Attentional focus (Action focused, Object focused) as between-subjects factor and Types of photo (backward, Forward 1, Forward 2, Forward 3) as within-subject factor on log transformed RTs. Given the null effect of the Type of action (Return vs. No return) in Experiment 1, we removed it from the model.

We found a main effect for Types of photo (*F*(3,270) = 17.84, *p* < 0.001, *η*_p_^2^ = 0.16), but not for Attentional focus (*F*(1,90) = 0.29, *p* = 0.59, *η*_p_^2^ = 0.003). In addition, a significant interaction was found between Attentional focus and Types of photo (*F*(3,270) = 3.78, *p* < 0.05, *η*_p_^2^ = 0.04).

To investigate this significant interaction, we conducted two repeated measures ANOVAs with Types of photo as within-subjects factor in each group (action focused, object focused) on RTs. In the action-focused group, we found a significant effect of the Types of photo (*F* (3,129) = 14.16, *p* < 0.001, *η*_p_^2^ = 0.25) and post hoc comparisons (Bonferroni adjusted) revealed significant differences between the evaluation of backward photos and all types of forward photos. RTs for backward photos were slower compared to Forward 1 photo (*p* < 0.001, CI [0.041, 0.11], Cohen’s *d* = 0.91), Forward 2 photo (*p* < 0.001, CI [0.018, 0.088], Cohen’s *d* = 0.63) and Forward 3 photo (*p* < 0.001, CI [0.036, 0.115], Cohen’s *d* = 0.85). The comparisons between the forward photos were not significant (p varied from 0.58 to 1, BF_01_ varied from 1.60 to 6.01).

Thus, the advantage in the evaluation of forward photos occurs for every type of forward photos, regardless of the distances between those forward photos and the photo presented at encoding.

In the object-focused group, we also found a significant effect of the Types of photo (Greenhouse–Geisser correction, *F*(2.35,110.672) = 4.56, *p* < 0.01, *η*_p_^2^ = 0.09), but post hoc comparisons (Bonferroni adjusted) revealed a significant difference only between the backward photos and the forward three photos (*p* < 0.001, CI [0.013, 0.064], Cohen’s *d* = 0.61), whereas the other comparisons were not significant (*p* value ranging from 0.09 to 1, BF_01_ varied from 0.36 to 5.26). When participants were asked to focus their attention on the object, they were faster in the evaluation of photos representing the last step of the action and consequently the last position of the object involved in the action (Forward 3 photo) compared to the backward photo. This advantage did not occur for the other types of forward photos.

No differences were detected between the two groups of participants’ RTs on each type of photo. We did not find a difference in the RTs for backward photos (*t*(90) = 0.52, *p* = 0.61, CI [− 0.517, 0.301], BF_01_ = 4.11), for Forward 1 photos (*t*(90) = 1.44, *p* = 0.15, CI [− 0.112, 0.711], BF_01_ = 1.77), for Forward 2 photos (*t*(90) = 0.37, *p* = 0.72, CI [− 0.332, 0.486], BF_01_ = 4.29) and for Forward 3 photos (*t*(90) = 0.74,* p* = 0.46, CI [− 0.255, 0.565], BF_01_ = 3.52). Table 3 shows the participants’ accuracy rates based on the Types of photo and the Attentional focus factors.

**Table 3:** Mean accuracy (and standard deviations) based on both Types of photo and Attentional focus factors in Experiment 2

Attentional focus	Types of photo			
	Backward	Forward 1	Forward 2	Forward 3
Object focused	0.97 (0.08)	0.98 (0.05)	0.99 (0.03)	0.99 (0.03)
Action focused	0.96 (0.08)	0.99 (0.04)	0.97 (0.06)	0.97 (0.06)

The assumptions of linearity and normality were difficult to assess reliably for the accuracy rate. We therefore performed an additional sensitivity analysis to account for within- and between-subject factors. We used the RM function in the MANOVA.RM (Friedrich et al., 2018) package in R. We extracted the ANOVA-type statistic (ATS) with the corresponding *p* values, as this test should work well even with violations of the normality assumption (Friedrich et al., 2018). We found no effect of the factors Attentional focus (ATS = 2.4, *p* = 0.12) or Types of photo (ATS = 12.57, *p* = 0.07). We also found no significant interaction between these two factors (ATS = 1.56, *p* = 0.21). Thus, participants in the object-focused and action-focused groups did not differ statistically in their accuracy in recognizing each type of photo.

## Discussion

The aim of the investigation was to shed light on the role played by participants’ attention on the object’s physical properties in action anticipation when we observe an action on an object. The innovative aspect of the present investigation is thus twofold. While Ianì et al., (2021, 2023) showed that the representation of the observed action unfolding in time speeds up the recognition of the next two action states, in Experiment 1 we thoroughly investigated the anticipation effects by testing performance in three different forward conditions instead of two. This approach allows us to examine the forward effects at a greater temporal distance and thus gain a deeper understanding of the unfolding in time of the action simulation. Moreover, Experiment 2 was devised to investigate the role of participants’ attention to the object in action anticipation. We manipulated participants’ attentional focus to determine the possible modulatory effect on the process of action anticipation triggered by the object of the observed action.

In Experiment 1, we extracted five equally spaced images in time from each of a series of action videos and showed participants the second image as the stimulus, followed by one of the following three images (forward photos) or the previous image (backward photo). The participants’ task was to judge whether the second photo depicted an action congruent with the action depicted in the first photo, i.e., whether the general action in the two photos was the same or whether it was different. As the results showed, all forward photos were judged faster than the backward photo. It is worth noting that the fifth photo (Forward 3 photo) was judged faster than the backward photo, although the backward photo was closer in time to the original stimulus.

Some actions that we used as experimental material involved not only the acquisition of an object for use but also an accompanying reverse action involving the return of the object, while other actions instead involved no return phase involving the same objects. Statistical analyses revealed no differences in accuracy or response time between the scenarios with and without return. This result is in line with the literature on the role of kinematics cues in predicting actions’ goals (in our cases, the goal to acquire or to returning the objects) discusses in the introduction. This is particularly relevant to our study because we took “full-body” screen captures, which provide participants with also important information from gaze direction (e.g., Ambrosini et al., 2015). For example, when the actress picks up the hamburger from the plate to eat, her gaze is directed to the point where he bites into the hamburger, whereas when she returns the hamburger, she looks at the plate to lean on the hamburger. Similarly, the grasping motion when picking up is different from the motion when returning. In addition, objects can also convey important information about the direction of the action. For instance, if a hamburger held in the hands is whole, the images are likely not depicting its return to the plate.

In Experiment 2, participants’ task was the same as in Experiment 1, but we additionally manipulated their attentional focus: half of the participants judged the congruence of the second photo with the first photo while focusing on the action, and half of the participants while focusing on the object. Participants who focused on the action evaluated all forward photos faster than the backward photo, whereas participants who focused on the object evaluated only the most forward photo faster than the backward photo. The results support the hypothesis that knowledge of the object involved in the action modulates action anticipation: people anticipate the actions of others by applying their motor schemas to the observed actions, but their knowledge about objects allows them to reason about the attainable goal given certain physical features of the object. The results confirm the prediction that focusing on the object modulates the process of action anticipation by modulating the mental simulation of the action. These results cannot be explained by the fact that the photos show the same objects: to make this explanation plausible, participants would have to spend a comparable amount of time judging the congruence of backward and forward photos with the photo presented during encoding, since all photos show the same object.

The idea that action anticipation can be modulated by participants’ attentional focus is consistent with the assumption that action identification should be separated from goal identification (Thompson et al., 2019). Action identification can be conceptualized as the ability to distinguish the observed action from others by using the intrinsic “configural relationship between body parts” (ibidem, p.107), without the need for inferential processing, and to use this motor information (“how” an action is performed) to interact effectively with others. Goal identification can instead be conceptualized as the ability to directly understand the goal of others’ actions (“why” an action is performed) via an inferential mechanism. Consistent with the proposed view, mirror neurons seem to be mainly involved in identifying actions, whereas the area PF (an area within the IPL) seems to be involved in identifying goals (see Osiurak et al., 2020). On this view, mirror activations could be responsible for action identification, but they should play a less crucial role in action anticipation at the goal level. As Osiurak and Reynaud (2020) suggest: “tool-use action anticipation might be much more disembodied as commonly assumed” (p. 230). This process could play a crucial role, especially when, as in our studies, manipulable artifacts were used as stimuli. As Frey (2007) noted, although the use of very simple tools (e.g., a rake) can directly alter sensorimotor activations, more complex manipulable artifacts (such as a cup, a screwdriver, or a knife) also require access to higher cognitive functions (i.e., semantic representations of object functions and uses). Osiurak et al. (2017) highlighted how simple objects represent affordances by directly exploiting the plasticity of human sensorimotor mechanisms, whereas more complex objects require knowledge about how the objects can effectively interact with other objects (Osiurak et al., 2017). The observation of actions involving objects provides two different kinds of information: the motor action that connects the model’s hand to the tool and the mechanical features of the objects that inform us about the possible goal of the action. By this, we do not mean to say that observing graspable or manipulable objects does not require the involvement of motor schemas as suggested by embodied theories (e.g., Barsalou et al., 2003). Rather, in addition to motor activations (e.g., Tucker & Ellis, 1998), observing objects triggers inference about the physical properties of the object, which can directly inform us about the possible goal of the action (see Osiurak et al., 2016; Osiurak & Reynaud, 2020). Understanding the tool use actions of others also requires additional cognitive abilities that are “neither based on motor information/programs nor on the activity of mirror neurons” (Osiurak et al., 2020; p.230).

Although we did not directly test the motor involvement in our experiments, the results of the present investigation might be relevant to the debate around the functional role of motor activation during action observation. Some authors interpret mirror activations as those at stake in simulating and thus in predicting the observed action (see, e.g., Gallese, 2007), while others consider them merely a first step on the way to a full mental representation of others’ actions (e.g., Goldman, 2009). It is surprising that these completely different views are often grouped under the same generic term “action anticipation”. This great uncertainty is probably due to the difficulty of delimiting and defining the nature of the mental processes which are based on these activations. In this view, mirror neurons are probably mainly involved in emulating and predicting step by step the observed movements rather than in directly represent the goal (Wilson & Knoblich, 2005; see also Pezzulo et al., 2013). Mirror activations would serve to establish the possible motor programs involved in the observed action and then predict the immediate kinematic consequences, whereas IPL and PF activations would serve guiding goal understanding. This interpretation would fit with a study in which participants viewed static photos depicting the beginning, the middle, or the end of actions and found that motor activation was maximal when the photos depicted incomplete actions rather than when they depicted the final states of the actions (e.g., Urgesi et al., 2010).

However, it is worth noting that similar forward effects have been also detected using 2D figures as stimuli instead of human movement. For example, in the work of Freyd and Finke (1984), participants were shown a static 2D rectangle in three orientations along a possible rotation path (each orientation was separated in time by an interstimulus interval (ISI) of 250 ms). Their task was to remember the last orientation of the sequence. They were then presented with a rectangle that was either the same or different from the third. If it was different, the rectangle could have two new orientations: a small rotation in the same direction as the implicit movement or an equally small rotation in the opposite direction. Again, it was more difficult for participants to reject rectangles with an orientation in the direction of the implicit motion, suggesting that their memory for the final image is systematically distorted along this direction, even in the case of no-conspecifics movements. In light of this literature, one could argue that this type of effect may just reflect “learned associations related to time”. The fact that similar forward effects are also detectable in non-human movements and can be interpreted as the result of learned associations acquired over a lifetime is also consistent with the associative interpretation of mirror neurons. Mirror neuron activation has been interpreted in several theoretical accounts as a type of simple association mechanism (for a review, see Heyes & Catmur, 2022). For example, Heyes (2001) argued that mirror neurons get their mirror properties through the standard mechanisms of sensorimotor associative learning. Initially, they are just motor neurons, i.e., they are active only during the execution of actions, and then they acquire their matching properties through the correlated experience of seeing and performing the same actions in everyday situations. In this view, even the development of mirror neurons is a kind of procedural learning. In other words, the matching properties of mirror neurons emerge through associative learning during individual development (Catmur et al., 2016). This “associative perspective” states that the “sensorimotor learning that builds mirror neurons is of exactly the same kind as the learning that produces Pavlovian and instrumental conditioning” (Heyes & Catmur, 2022; p.159). Indeed, simulations arising from action observation are modulated by the observer’s expertise and prior experience (Calvo-Merino et al., 2005): neural activation during action observation is greater when participants are familiar with the observed action compared to neural activation arising from unusual actions or actions outside the reach of ordinary human motor skills. According to this view, the simulation triggered by action observation is also essentially a procedural memory process (Ianì et al., 2020). Further neurocognitive studies using the same paradigm (e.g., in conjunction with rTMS) are needed to explore more in depth the role of the mirror system.

## Conclusions

The innovative purpose of our experiments was twofold. First, in Experiment 1, we used three different types of forward photos, rather than two as in Ianì et al. (2023), to test the “length” of the forward effect (i.e., whether the advantage for forward photos manifests only at a very close action phase or even at the action target). Second, in Experiment 2, we also aimed to show how different forward effects could arise that were the result of different cognitive mechanisms by manipulating participants’ attentional focus. In other words, we aimed to demonstrate that the mechanism of action anticipation is not a unitary and homogeneous phenomenon, but can be based on different cognitive processes that use different kinematic and environmental cues.

The results of the present investigation argue for an action prediction mechanism by which people simulate several different next states of the observed action on objects through a kinematic mental simulation. In Experiment 1, when participants saw a still photo of an action on an object followed by photos depicting states of the action yet to come, they were quicker to judge the action as congruent with the photo they originally saw, compared with photos showing a state of the action that preceded the one they observed.

The results also suggest that the process can be influenced by knowledge of the physical properties of the object in question. When participants in Experiment 2 performed the same task but focused on either the action or the object, the results for the action-focused group replicated those from Experiment 1, but the results for the object-focused group showed that judgments were faster only for the state to come farther. Overall, these results suggest that knowledge about the object plays a specific role in anticipating actions on objects.

## Data Availability

The Supplementary Materials, Matlab script, and the data reported in this paper are publicly available at the Open Science Framework website: https://osf.io/kytrm/.

## Appendix A

See Table 4.

**Table 4:** The description of the action performed by the actress in each video (Experiments 1 and 2)

Video number	Description	Type of action	
1	Peel a banana	NR	
2	Drink a glass of water	R	
3	Put on a hat	R	
4	Blow the nose	NR	
5	Sniff a flower	R	
6	Tear a sheet of paper	NR	
7	Eat a hamburger	R	
8	Open a book	R	
9	Use eye drops	R	
10	Close a portable computer	R	
11	Cut a pizza	NR	
12	Make a tea	NR	
13	Smoke a cigarette	NR	
14	Wear eyeglasses	R	

Videos from 1 to 7 were used as target stimuli, whereas videos from 8 to 14 were used as filler at test. *R* means return actions, *NR* means no-return actions

## Footnotes

1 We also report Bayes factors to determine the relative probability of observing the present data under the null hypothesis compared with the alternative hypothesis (see Rouder et al., 2009). Bayes factor tests were run using JASP software (JASP Team, 2017) with a default JASP prior, i.e., a Cauchy prior with a location parameter of 0 and scale parameter of 0.707. Evidence in favour of the null hypothesis is denoted BF_01._
